# Graphene Oxide-Linezolid Combination as Potential New Anti-Tuberculosis Treatment

**DOI:** 10.3390/nano10081431

**Published:** 2020-07-22

**Authors:** Flavio De Maio, Valentina Palmieri, Giulia Santarelli, Giordano Perini, Alessandro Salustri, Ivana Palucci, Michela Sali, Jacopo Gervasoni, Aniello Primiano, Gabriele Ciasca, Maurizio Sanguinetti, Marco De Spirito, Giovanni Delogu, Massimiliano Papi

**Affiliations:** 1 Dipartimento di Scienze di Laboratorio e Infettivologiche, Fondazione Policlinico Universitario “A. Gemelli” IRCSS, 00168 Rome, Italy; flavio.demaio@unicatt.it (F.D.M.); ivana.palucci@unicatt.it (I.P.); michela.sali@unicatt.it (M.S.); maurizio.sanguinetti@unicatt.it (M.S.); 2 Dipartimento di Scienze biotecnologiche di base, cliniche intensivologiche e perioperatorie—Sezione di Microbiologia, Università Cattolica del Sacro Cuore, 00168 Rome, Italy; giulia.santarelli@gmail.com (G.S.); alessandro.salustri@unicatt.it (A.S.); giovanni.delogu@unicatt.it (G.D.); 3 Dipartimento di Neuroscienze, Università Cattolica del Sacro Cuore, 00168 Roma, Italy; giordano.perini@unicatt.it (G.P.); gabriele.ciasca@unicatt.it (G.C.); marco.despirito@unicatt.it (M.D.S.); 4 Istituto dei Sistemi Complessi, CNR, Via dei Taurini 19, 00185 Roma, Italy; 5 Fondazione Policlinico Universitario “A. Gemelli” IRCSS, 00168 Rome, Italy; jacopo.gervasoni@policlinicogemelli.it (J.G.); aniello.primiano@unicatt.it (A.P.); 6 Dipartimento di Scienze Biotecnologiche di Base, Cliniche Intensivologiche e Perioperatorie, Università Cattolica del Sacro Cuore, 00168 Roma, Italy; 7 Mater Olbia Hospital, 07026 Olbia, Italy

**Keywords:** graphene oxide, tuberculosis, trapping, nanotechnology, linezolid, therapy

## Abstract

Global pandemic management represents a serious issue for health systems. In some cases, repurposing of existing medications might help find compounds that have the unexpected potential to combat microorganisms. In the same way, changing cell–drug interaction by nanotechnology could represent an innovative strategy to fight infectious diseases. Tuberculosis (TB) remains one of the most alarming worldwide infectious diseases and there is an urgent need for new drugs and treatments, particularly for the emergence and spread of drug-resistant *Mycobacterium tuberculosis* (*Mtb*) strains. New nanotechnologies based on carbon nanomaterials are now being considered to improve anti-TB treatments, and graphene oxide (GO) showed interesting properties as an anti-TB drug. GO, which preferentially accumulates in the lungs and is degraded by macrophagic peroxidases, can trap *Mycobacterium smegmatis* and *Mtb* in a dose-dependent manner, reducing the entry of bacilli into macrophages. In this paper, combinations of isoniazid (INH), amikacin (AMK) and linezolid (LZD) and GO anti-mycobacterial properties were evaluated against *Mtb* H37Rv by using a checkerboard assay or an in vitro infection model. Different GO effects have been observed when incubated with INH, AMK or LZD. Whereas the INH and AMK anti-mycobacterial activities were blocked by GO co-administration, the LZD bactericidal effect increased in combination with GO. GO-LZD significantly reduced extracellular mycobacteria during infection and was able to kill internalized bacilli. GO-LZD co-administration is potentially a new promising anti-TB treatment at the forefront in fighting emerging antibiotic-resistant *Mtb* strains where LZD administration is suggested. This innovative pharmacological approach may lead to reduced treatment periods and decreased adverse effects. More importantly, we demonstrate how nanomaterials–drugs combinations can represent a possible strategy to quickly design drugs for pandemics treatment.

## 1. Introduction

Carbon nanomaterials (CNMs), such as graphene, graphene oxide, graphene quantum dots, carbon nanotubes and many others, have been widely investigated for biomedical applications, from theranostic to prosthesis bio-printing [1,2,3,4].

In the field of microbiology, it has been largely demonstrated that the CNMs’ unique structural and chemical properties can be exploited to change interactions between cells and microorganisms. In particular, graphene oxide (GO), the bidimensional carbon allotrope enriched in oxygen functionalities, interacts and disrupts bacteria structures, and it has been proposed as an antibacterial nanomaterial, both for medical device surface coating and systemic administration purposes [4,5,6,7,8].

The antimicrobial effects of GO are, however, largely driven by its size and oxidation degree, by the ionic strength and salinity of the environment and by microorganisms’ size and shape. Our group has demonstrated that in the presence of divalent cations, or in a highly nutrient growth medium, GO instability causes the inhibition of the so-called “blade effect” of thin GO sheets and the lack of destruction of bacterial membranes [9]. With ions in solution, GO creates a trapping net on which the bacteria can grow or stay physically stacked remaining alive [6].

Recently, we examined the effects of GO trapping on *Mycobacterium tuberculosis* (*Mtb*), a large rod-shaped bacterium and the etiologic agent of human tuberculosis (TB) [10]. During infection, *Mtb* is phagocytized by alveolar macrophages, where it can replicate and infect nearby cells and then spread to other tissues [11]. GO traps *Mtb* extracellularly thereby reducing entry in macrophages [10]. This is of particular interest considering also the GO lung targeting ability when injected intravenously [12]. Extracellular trapping could potentially improve the efficacy of the anti-TB treatment, due to the pharmacodynamic of some important anti-TB drugs, thereby enhancing anti-TB activity, which in turn may reduce the risk of toxicity and emergence of bacterial resistance.

For these reasons, new therapeutic delivery devices are urgently needed for TB treatment [13], and GO composites could potentiate the efficacy of anti-TB drugs, as already demonstrated for other antimicrobial compounds [4,7].

In this work, we investigated the effects of GO–drug combinations to develop an effective treatment. We combined GO with isoniazid (INH), amikacin (AMK) and linezolid (LZD) to investigate whether the effects with GO can enhance the *Mtb* killing efficacy. INH, one of the most powerful anti-TB drugs, is a prodrug that enters in the mycobacterial cell by passive diffusion and is activated by the mycobacterial catalase KatG [14]. AMK, an aminoglycoside antibiotic, is a second-line drug against TB and it is commonly used to treat non-tuberculous mycobacteria (NTM) infections. LZD, commonly used to treat bacterial pneumoniae, has been introduced as a second-line drug in the treatment of multidrug-resistant (MDR) or extensively drug-resistant (XDR) TB [15,16,17].

In this study, we show how the physical interaction between each drug and the GO surface determines the efficacy of the combined treatment. In axenic culture, we show how AMK and INH interact strongly with GO flakes, losing their efficacy. Conversely, LZD that is poorly adsorbed on GO surface remains effective and its ability to kill *Mtb* is increased in the presence of GO. We analyzed the mechanism of LZD-GO synergic effect in an in vitro infection model highlighting that GO strengthens the LZD activity against *Mtb*. Our results can be exploited for the design of innovative and more effective GO-based anti-TB therapies.

## 2. Materials and Methods

### 2.1. GO Characterization

GO (4 mg/mL) was purchased from GrapheneA (Spain). GO size was evaluated by atomic force microscopy (Nanowizard II JPK Instruments AG, Berlin, Germany) as previously reported [7] and GO hydrodynamic properties were measured by dynamic light scattering (DLS) using Zetasizer Nano ZS (Malvern, Herrenberg, Germany), as previously reported [18]. Drug-conjugated GO was prepared using a fixed GO concentration and incubating with drug (AMK, INH or LZD) diluted in ultrapure water. Anti-TB drugs were used at twice the MIC concentration (2 μg/mL for AMK or LZD and 0.4 μg/mL for INH, respectively) and at excess of the MIC point established in 60 μg/mL. Drugs and GO were allowed to interact for 15 min and then the unbound drug was eliminated by resuspending GO–drug pellets after centrifugation (14,000 g, 10 min) in fresh water. Centrifugation was repeated 2 times. The GO samples and GO drug-conjugated samples were investigated using attenuated total reflectance-Fourier transform infrared spectroscopy (ATR-FTIR) with the Spectrum One spectrometer (Perkin Elmer, USA).

The material under investigation was directly laid upon the ATR crystal and the spectra were recorded in the wave number range of 4000–5500 cm^−1^. Spectra were reported according to the literature [19].

### 2.2. Bacterial Manipulation

Each experiment was performed by using *Mycobacterium tuberculosis* (*Mtb*) reference strain *Mtb* H37Rv expressing the green fluorescent protein (GFP) under the control of the mycobacterial hsp60 gene promoter (*Mtb-*GFP) [20]. Experiments were carried in biosafety level 3 (BSL3) laboratory. Bacteria were grown in 7H9 broth medium (Difco) enriched with 10% albumin dextrose catalase (ADC) (Sigma-Aldrich) and 0.05% Tween 80 (Sigma-Aldrich, Germany) supplemented with 50 μg/mL hygromycin, at 37 °C and 110 rpm agitation, until a concentration of OD600 between 0.5 and 0.8. Bacterial cell culture was added with 20% sterile pure glycerol (Carlo Erba Reagents, Italt) and stored at −80 °C.

### 2.3. In Vitro Antimicrobial Assay

The antimicrobial assays were executed by incubating *Mtb-*GFP with a scalar concentration of graphene oxide (GO) from 0.5 to 0.05 mg/mL supplemented with anti-mycobacterial drugs: isoniazid (INH), amikacin (AMK) and linezolid (LZD). Anti-mycobacterial drugs were added alone or in combination with GO starting from minimal inhibitory concentrations in a homemade checkboard assay. Each experiment was carried out in 7H9 medium enriched with 10% ADC and 0.05% Tween80, as described above. Bacteria were seeded in a sterile 96 multi-well plate and incubated at 37 °C, for 15 days [21]. After incubation, cell viability was assessed by measurement of the GFP emission fluorescence and measurement of turbidity at OD 600nm. Fluorescence intensity was then quantified by using ImageJ software [10,22]. To corroborate previous results, each well that was identified as the MIC point was plated on 7H11 medium supplemented with 10% OADC.

### 2.4. Cell Culture

Murine macrophages (J774 A.1 cell line) were cultured in Dulbecco’s modified Eagle’s medium (DMEM) (Euroclone) supplemented with 10% inactivated fetal bovine serum (FBS) (Euroclone, Italy), 1% L-glutamine (Euroclone) and 1% streptomycin–penicillin (Euroclone) and were incubated at 37 °C and 5% CO_2_. Adherent cells were washed with sterile warm phosphate buffered saline (PBS) (Euroclone) and removed for experiments by using 1× trypsin in PBS (Euroclone). Cells were counted and re-suspended in DMEM supplemented with 2% FCS and 1% L-glutamine. Finally, cells were seeded in sterile 48 well plates (Euroclone) at a concentration of 1.2 × 10^6^ cells/well and incubated overnight until infection or treatment [10,23].

### 2.5. Mycobacterial Infection

J774 cells were infected with *Mtb-*GFP, with multiplicity of infection (MOI) of 1 (1 bacterium to 1 cell) in two different ways. In the first experiment, plated cells were infected with a medium containing bacteria alone; bacteria and GO at the concentrations of 0.25 mg/mL; bacteria and LZD at the concentrations of 1, 0.1, and 0.01 μg/mL; or bacteria and a combination of GO and LZD at the concentrations previously indicated. Infected cells were incubated at standard atmosphere conditions. One hour (h) post-infection, infected cells were washed two times with sterile warm phosphate buffered saline (PBS) (Euroclone) in order to remove extracellular bacteria, and a new sterile medium was added for 4 h. Finally, colony forming units (CFUs) were performed by harvesting infected cells with sterile 0.1 mL of sterile Triton X-100 (Sigma-Aldrich). Serial dilutions were performed before plating on 7H11, containing 10 OADC. Plates were incubated at 37 °C for 15 days. In the second experimental setting, cells were infected with *Mtb-*GFP alone for 1 h. Four hours after the infection, a new medium containing GO, LZD or a combination of GO/LZD as previously mentioned was added and incubated for 24 h when CFU_S_ were performed.

### 2.6. LDH and ROS Detection

To measure cell viability and reactive oxygen species induced by GO treatment, murine macrophages (J774) were cultured as previously described and then plated at the final concentration of 1.2 × 10^6^ cell/mL in a 96-well plate. J774 cells were incubated overnight at standard atmosphere conditions (37 °C and 5% CO_2_), until they were incubated with GO alone at concentrations ranging from 0.06 to 0.25 mg/mL, LZD (at final concentrations of 1, 10, and 100 µg/mL) and combinations of 0.25 mg/mL GO and LZD at the indicated concentrations. Cells were incubated 24 h until lactate dehydrogenase (LDH) and ROS were measured.

Briefly, LDH was evaluated on the supernatants of treated cells opportunely centrifuged to remove GO in the solution. Each supernatant was diluted before incubation with the substrate. Thirty minutes later, absorbance at 450 nm was measured.

For the detection of ROS, the fluorinated derivative of 2′,7′-di-chlorofluorescein (H_2_DCFDA) was employed. This probe is non-fluorescent until the acetate groups are removed by intracellular esterases and oxidation occurs within cells. Thus, oxidation can be detected by monitoring the increase in fluorescence intensity. After the treatment, the medium was removed, and cells were carefully washed with warm sterile PBS. Then, cells were treated with PBS containing 10 μM H_2_DCFDA and incubated for an additional hour at 37 °C, in 5% CO_2_. PBS containing H_2_DCFDA was finally removed and a new medium was added. Fluorescence intensity of H_2_DCFDA was recorded by using a Cytation 3 Cell Imaging Multi-Mode Reader by exciting at 495 nm and recording emission at 528 nm.

## 3. Results

### 3.1. Characterization of GO-Drugs Interaction

**Figure 1:**
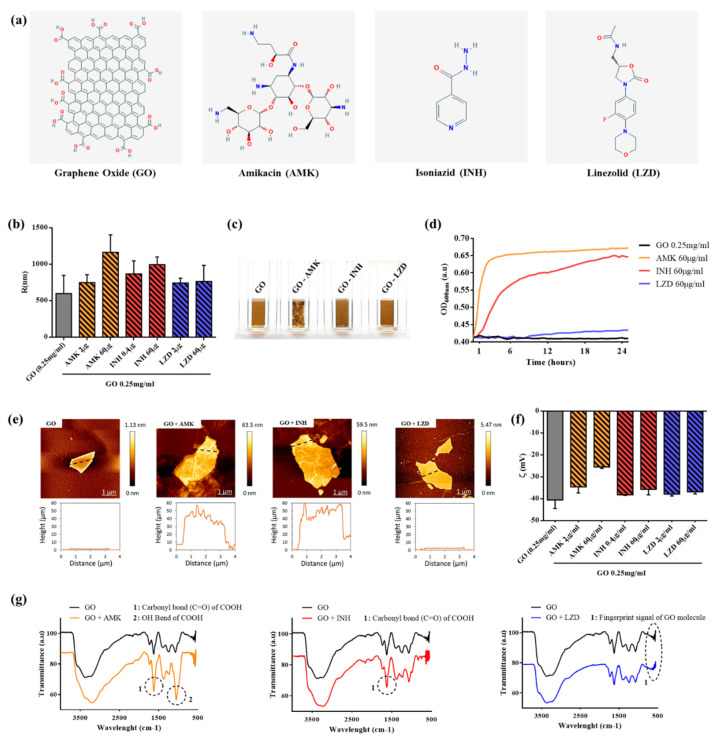
Characterization of GO interaction with AMK, INH and LZD. (**a**) Chemical structure of graphene oxide (GO) and drugs used in this study (AMK, INH, LZD). (**b**) The hydrodynamic radius of GO flakes alone or in presence of different concentrations of drugs measured by dynamic light scattering. (**c**) Photograph of cuvettes containing different GO–drug samples, visible aggregates are present with AML and INH. (**d**) Optical density evaluation of sample stability over time, the increase in the OD signal is due to aggregation of GO-AMK and GO-INH samples. (**e**) Representative atomic force microscopy micrographs of GO flakes and their corresponding height profiles. (**f**) Zeta potential of different samples. (**g**) FTIR characterization of sample’s surface with clear differences of GO-AMK and GO-INH compared to bare GO. GO-LZD does not show significant differences from GO alone. FTIR spectra of drugs alone are reported for comparison.

In Figure 1a, we report the chemical structure of graphene oxide (GO) and drugs used for this study, amikacin, isoniazid and linezolid. GO flakes used in our work had a mean hydrodynamic radius of ~600 nm (Figure 1b). We incubated GO with the different drugs to allow adsorption on the surface and we obtained three GO–drug formulations: GO with amikacin (GO-AMK), GO with isoniazid (GO-INH) and GO with linezolid (GO-LZD). The unbound drug was eliminated by several washing steps.

We used several ratios of GO–drug for the characterization of interactions. We observed that immediately after incubation, GO-AMK formed visible large aggregates (Figure 1c) and that small aggregates were also visible in the GO-INH sample. GO-LZD remained homogeneous, even after ~17 h, when GO-INH and GO-AMK precipitated. The immediate increase in size after GO-AMK interaction was also recorded by dynamic light scattering (DLS) and was less pronounced for GO-INH. GO-LZD did not show a significant increase in hydrodynamic radius (Figure 1b). We measured that in a time of 25 h, GO and GO-LZD remained stable in solution without an appreciable increase in OD, while GO-AMK showed an immediate OD increase and reached a plateau after ~2 h (Figure 1d). Similarly, GO-INH aggregated in solution and reached an OD plateau after ~12 h, with a slower increment.

To investigate GO–drug interactions, we characterized samples in terms of morphology (by atomic force microscopy), surface zeta potential and IR absorption. Representative AFM images and corresponding line profiles of GO, GO-AMK, GO-INH and GO-LZD are reported in Figure 1e. Profiles clearly show how AMK and INH are deposited on GO surface, while LZD does not interact with GO. Zeta potential measurements confirm AFM results (Figure 1f) for LZD, which did not affect the surface potential of GO, even at a high concentration (60 µg). AMK, conversely, modified the surface potential of GO, making it less negative and consequently more prone to aggregation, even at a low concentration (2 µg). INH had an effect on GO surface charge only at a high concentration (60 µg) and to a lesser extent compared to AMK.

In Figure 1g, ATR-FTIR spectra of GO, GO-AMK, GO-INH and GO-LZD are shown. The IR spectrum of GO shows the characteristic IR absorption bands: the strong broad band at 3400 cm^−1^ corresponds to the O-H stretching vibration, the band at 2923 cm^−1^ is attributed to the vibration of C–H bond, the band at 1731 cm^−1^ corresponds to the stretching of the carboxylic groups (C=O), the peak at 1624 cm^−1^ is attributed to C=C stretching, the band at 1394 cm^−1^ corresponds to hydroxyl group deformation and the bands at 1235, 1069, and 987 cm^−1^ are characteristic of bending modes associated with oxygen functional groups.

The spectra clearly show differences in the IR profile in GO-AMK and GO-INH respect to GO alone, underlying the presence of a conjugation between drugs and GO. Precisely, INH and AMK interaction with GO was highlighted by changes in FTIR transmittance for carbonyl bonds of carboxyl groups (1690 cm^−1^) and O-H bonds (1395 cm^−1^). Interaction between GO and LZD was not identified by FTIR analysis, which also showed the same fingerprint region in both GO and GO-LZD spectra, suggesting the absence of drug conjugation [24,25].

### 3.2. Graphene Oxide Has an Additive Effect with Linezolid, but not with Isoniazid and Amikacin

**Figure 2:**
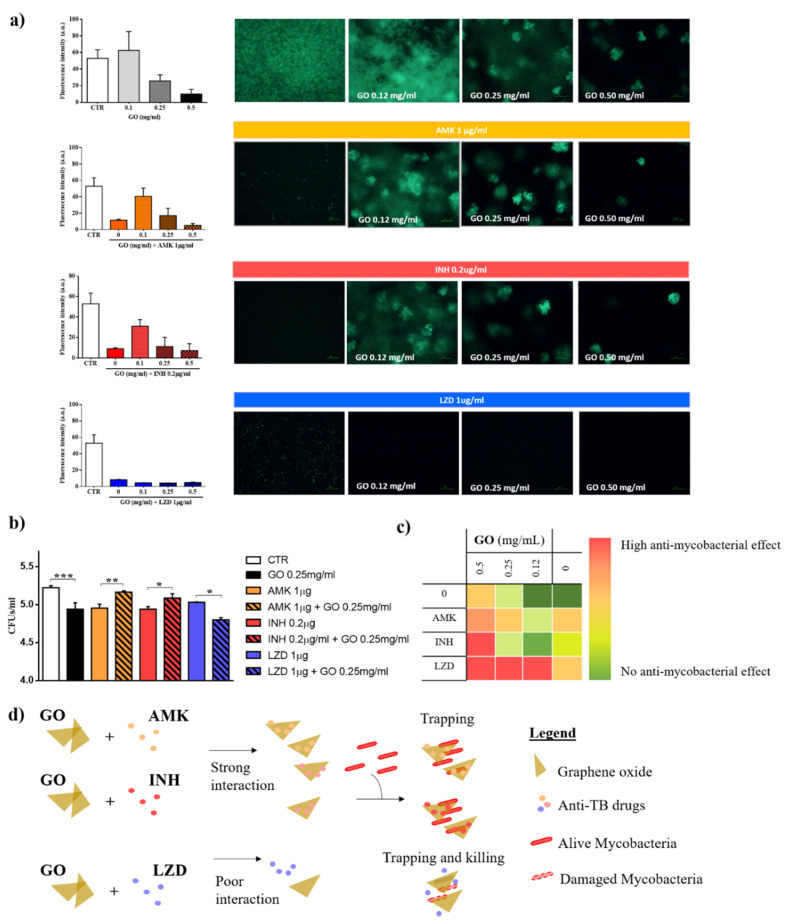
Evaluation of GO and GO-drugs formulations on *Mtb* H37Rv expressing the cytosolic GFP. (**a**) Measurement of fluorescence intensity after treatment with graphene oxide (GO), amikacin (AMK), isoniazid (INH) and linezolid (LZD) and representative images of mycobacteria after treatments. (**b**) Colony forming unit (CFU) analysis (logarithmic scale) after treatment with GO (0.25 mg/mL), AMK (1 μg/mL), INH (0.2 μg/mL) and LZD (1 μg/mL) or GO combined with AMK, INH or LZD at the previously indicated concentrations. (**c**) Scheme of the anti-mycobacterial effects of GO alone and in combination with drugs on *Mtb*, from lack of effect (green) to highest effect (red). (**d**) Schematic representation of the interaction between GO and drugs and its effects on mycobacterial trapping and killing. In each figure, *p* values have been indicated with the asterisk (*p* < 0.05 = *; *p* < 0.01 = ** *e*; *p* < 0.001 = ***).

We have previously reported GO activity against *Mycobacterium smegmatis* (*Ms*) and *Mtb* demonstrating that this nanomaterial entraps mycobacteria in an extracellular net, without affecting their viability [10]. To investigate GO effects in combination with different anti-TB, we set up a checkboard-based assay with different GO concentrations (0.5, 0.25, and 0.12 mg/mL), administered with AMK, INH and LZD at their minimal inhibitory concentrations (MIC) (1, 0.2, and 1 μg/mL, respectively). The combinations of the GO and selected anti-TB drugs were assayed against *Mtb H37Rv* reference strain expressing the cytosolic GFP. After 15 days, each well was analyzed, and images were acquired by using fluorescence microscopy (Figure 2a).

The GO trapping effect is enhanced with AMK and INH, as demonstrated by the reduced bacterial fluorescence and aggregation (0.5 and 0.25 mg/mL). The increased instability of GO in solution may explain the trapping effect. However, with the reduction in GO concentration at 0.1 mg/mL, the trapping effect/bactericidal activity of the INH and AMK was abolished. Conversely, when GO was co-administrated with LZD, bacterial fluorescence decreased at all concentrations and appeared slightly lower compared to LZD alone (Figure 2a–c).

To assess bacteria viability, GO combination with INH, AMK and LZD at their MICs were administered at decreasing concentrations in a *Mtb* axenic culture and colony forming units (CFUs) were determined four hours after treatment [10]. GO-LZD was confirmed to be the only combination capable of reducing CFUs compared to untreated *Mtb* culture, and to enhance anti-mycobacterial activity compared to the GO alone (Figure 2b). More importantly, CFU analysis confirmed that treatment with GO-AMK or GO-INH is not bactericidal and that drugs are not effective on GO surfaces. Figure 2c schematically illustrates the consequences on *Mtb* viability of the interactions of different GO-drugs formulations. Trapping is enhanced at high GO concentrations with all drugs; however, only LZD-GO formulation is always more effective than GO alone or LZD alone. Taken together, these results indicate that GO-AMK and GO-INH favor trapping without inhibiting bacteria viability, while GO appears to synergize with LZD, probably because LZD does not interact with the GO surface (see Figure 2d for a scheme of observed interactions).

**Figure 3:**
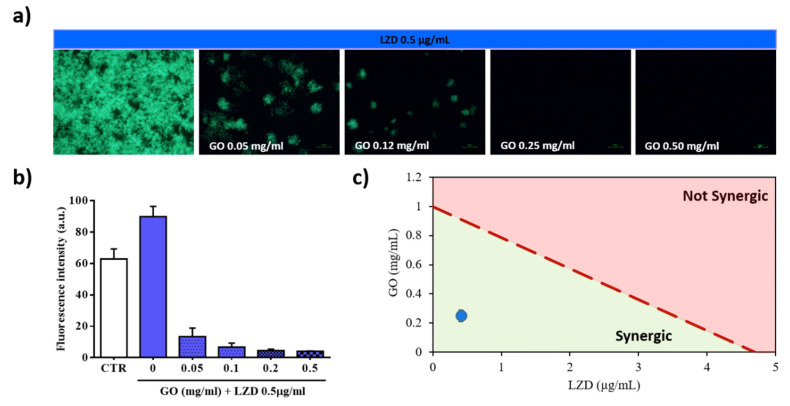
Evaluation of synergy between GO and LZD (MIC/50) *Mtb*-GFP viability assessed by measuring fluorescence intensity after treating the culture with GO, LZD (MIC/50: 0.5 μg/mL) and GO-LZD (MIC/50). Representative images of *Mtb*-GFP after treatment (**a**). Values of the average fluorescence intensities obtained from analyzed images (**b**) and evaluation of the agonist effect between LZD and GO by the construction of the isobole graph (**c**). The red dashed line connects MIC/50 concentrations of GO and LZD (**c**).

To assess the synergy between GO and LZD, we tested a combination of MIC/50 of the LZD and GO (0.25 mg/mL) (Figure 3). Administration of LZD alone on *Mtb*-GFP culture did not inhibit mycobacterial replication (Figure 3a,b). Remarkably, GO co-administration was confirmed to improve the efficacy of the LZD in a GO-concentration dependent manner. We then verified the synergy of the two molecules by creating isoboles graphs (Figure 3c). In these graphs, the concentrations of GO and LZD capable of reducing bacteria viability by 50% is connected by a dashed line. This line defines two areas in which there is an agonist effect (below the line) or an additive effect (above the line). The treatment is synergistic, as highlighted in the isoboles. These results suggest that co-administration of GO with LZD is endowed with synergistic anti-mycobacterial effect.

### 3.3. GO Co-Administration Enhances LZD Activity during the Infection of Macrophages

Given the proven efficacy of the combination GO-LZD, we tested the GO-LZD activity in an in vitro standard model of *Mtb* infection that can simulate early phases of infection (4 h) and later phases with early replication of the mycobacteria inside phagocytic cells (24 h).

**Figure 4:**
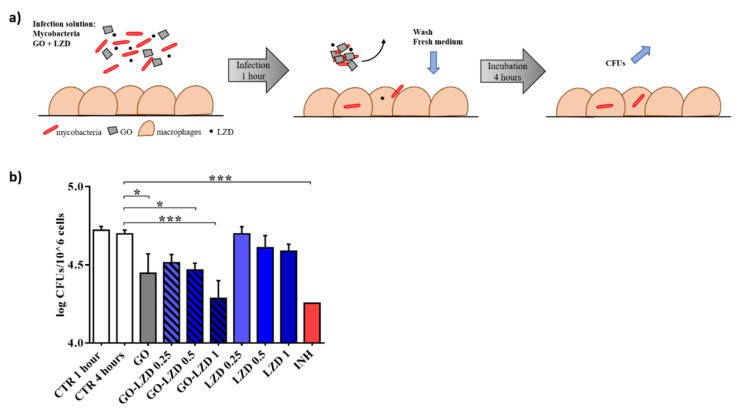
GO-LZD effects in the early phases of the *Mtb* infection. Murine macrophages (J774) were infected with *Mtb* (MOI = 1:1) in presence of GO (0.25 mg/mL), LZD (1, 0.5, and 0.25 μg/mL), GO-LZD combinations, and INH (0.2 μg/mL). One hour after the infection, cells were washed and new fresh medium was added. Four hours later, colony forming units (CFUs) were evaluated (**a**). CFUs were reported in log10 scale (**b**). In each figure, *p* values have been indicated with the asterisk (*p* < 0.05 = *; *p* < 0.01 = ** *e; p* < 0.001 = ***).

We have previously demonstrated that GO prevents mycobacterial entry in macrophages, generating a net that blocked *Ms* or *Mtb* in the extracellular space [10]. Mycobacteria entrapment outside of the host cells could play an important role to improve the efficacy of the anti-TB regimens. Besides the reduction in the number of infected cells, mycobacteria could be more easily reached by drugs in the extracellular compartment, especially given the little internalization of pharmaceutical compounds in the cells. We infected murine macrophages (J774) with *Mtb* adding GO (0.25 mg/mL) and LZD (1, 0.5, and 0.25 μg/mL) alone, or a combination of GO (0.25 mg/mL)—LZD (1, 0.5, and 0.25 μg/mL). INH (0.2 μg/mL) was used as a control. One hour after the infection, the medium was removed and J774 cells were washed and finally incubated for 4 h with fresh medium until CFUs were determined (Figure 4a).

CFUs decreasing when GO alone was added corroborated what previously demonstrated by our group confirming the entrapment of mycobacteria GO-dependent. As showed in axenic culture, LZD (MIC and MIC/50) was significantly capable of reducing CFUs in combination with GO (Figure 4b) compared to untreated cells or with mycobacteria treated with MIC, MIC/50 or MIC/25 concentrations of LZD alone. GO is able to maximize or to exert LZD efficacy up to level similar to INH. These data corroborated the synergic effect of the GO when co-administrated with LZD in the early phases of infection.

Subsequently, J774 were first infected with mycobacteria alone and new fresh medium was added one-hour post infection. This method allows unperturbed mycobacteria entry.

**Figure 5:**
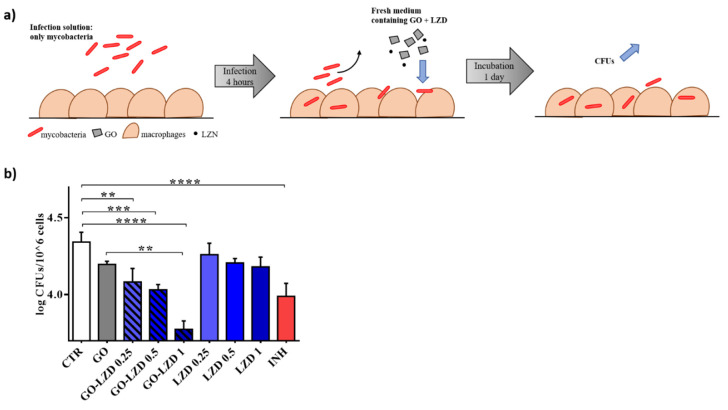
GO-LZD effects after 24 h since the *Mtb* infection. Murine macrophages (J774) were infected with *Mtb* (MOI = 1:1). One hour later, the infection solution was removed, cells were washed and new fresh medium was added for 4 h until GO (0.25 mg/mL), LZD (1, 0.5, and 0.25 μg/mL), GO-LZD combinations, and INH (0.2 μg/mL) were administrated. (**a**). Colony forming units (CFUs) were evaluated 24 h later and were represented in log10 scale (**b**). In each figure, *p* values have been indicated with the asterisk (*p* < 0.05 = *; *p* < 0.01 = ** *e; p* < 0.001 = ***; *p* < 0.0001 = ****).

At four hours post infection, GO (0.25 mg/mL) and LZD (1, 0.5, and 0.25 μg/mL), or a combination of GO (0.25 mg/mL) and LZD (1, 0.5, and 0.25 μg/mL) were added. INH (0.2 μg/mL) was used as a control. CFUs were evaluated 24 h after treatment (Figure 5a). Whereas no significant activity was observed when LZD was used to treat the infected cells, the co-administration of GO with LZD significantly reduced intracellular mycobacteria (Figure 5b) in an LZD concentration-dependent manner. Interestingly, while GO-LZD (0.5 μg/mL and 0.25 μg/mL) had an efficacy comparable to INH, GO-LZD at standard MIC point (1 μg/mL) reduced CFUs at values considerably lower than INH treatment. Notably, a reduction trend was also observed for GO alone. These results suggest that GO-LZD combination may exert an anti-mycobacterial activity also on intracellular mycobacteria, likely due to cell–nanomaterial interaction.

### 3.4. GO Affects Macrophages Permeability and ROS Production

GO showed a slight activity on intracellular bacteria; however, data on GO-LZD combination indicate that GO interacts with the eukaryotic cells and maximizes LZD internalization and/or affects the cellular antimicrobial properties.

**Figure 6:**
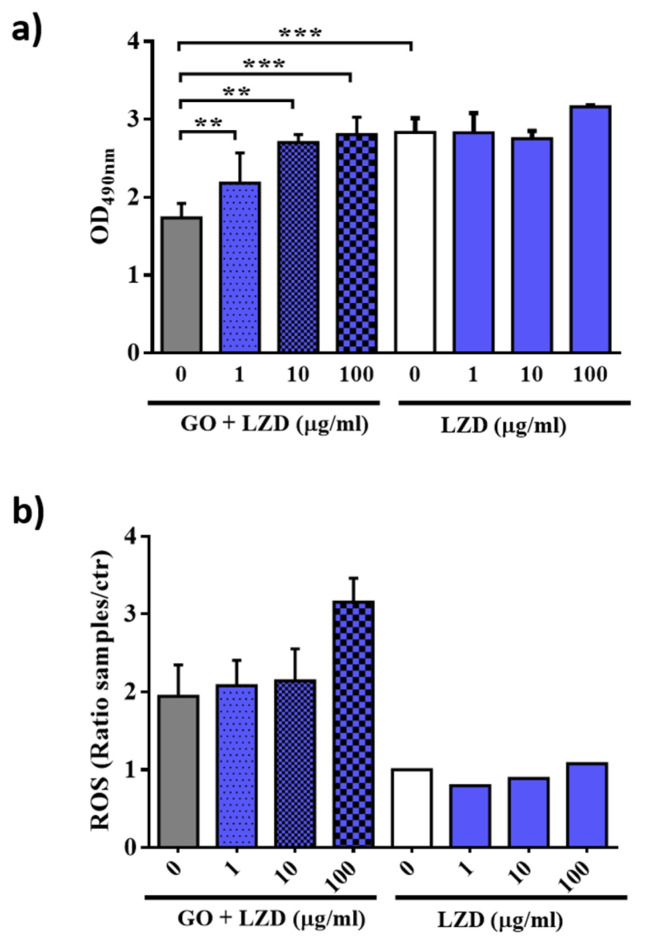
Cell viability and ROS production assessment after GO-LZD treatment. Quantification of cell viability by measuring lactate dehydrogenase (LDH) (**a**) and reactive oxygen species (ROS) production (**b**) after treatment with GO (0.25 mg 7ml), LZD (1, 10, and 100 μg/mL) alone or in combination with GO. Each experiment was performed on J774 murine macrophages and measures were carried out after 24 h of treatment. In each figure, *p* values have been indicated with the asterisk (*p* < 0.05 = *; *p* < 0.01 = ** *e; p* < 0.001 = ***)

To better understand the mechanism that allows the mycobacterial reduction in infected cells, we investigated cell viability and measured oxygen reactive species induction by GO, LZD or a combination of GO-LZD treatments (Figure 6). To maximize these effects, we treated macrophages (J774) with LZD 1 (standard MIC), 10, or 100 µg/mL.

For cell viability evaluation, lactate dehydrogenase (LDH) was measured in the supernatants of treated cells. When the cell membranes are compromised or damaged, LDH is released into the surrounding extracellular space. The treatment of LZD alone did not significantly change the viability of macrophages even at high concentration (Figure 6a). Similarly, GO-LZD did not cause cell toxicity, even at the highest concentration. Interestingly, with GO alone or GO-drug combination containing low LZD concentration the cells appeared more viable. This indicates that GO could induce cell proliferation.

Treatment of cells with LZD alone did not induce the production of reactive oxygen species (ROS) at all tested concentrations. Conversely, the combination of GO-LZD or GO alone determined a two to three-fold increase in the ROS amount (Figure 6b) compared to controls without nanomaterial. Taken together, our results underline that GO is not toxic on macrophages and that it can support LZD treatment in both extracellular milieu by trapping or when mycobacteria are intracellularly localized by stimulating ROS mediated bactericidal mechanism.

## 4. Discussion and Conclusions

Repurposing of existing medications in combination with nanomaterials might help find compounds that have the unexpected potential to combat *Mtb* and more generally all microorganisms responsible for pandemics.

Common anti-TB regimens rely on the combined use of drugs and last at least 6 months: isoniazid, rifampin, pyrazinamide and ethambutol are administered during the first 2 months, while only isoniazid and rifampicin are administered for the remaining four months. In the case of toxic events for a specific drug or during treatment of drug-resistant strains, regimens are adjusted to include second-line drugs, that are less effective and even more toxic, which further extends regimens’ duration and reduces success rate [26,27]. Novel delivery strategies, new drugs or drug combination with enhanced activity may lead to shorter regimens or improved outcomes. The use of CNMs in combination with first or second-line anti-TB drugs may enhance the activity of existing regimens. Among nanomaterials, GO shows important anti-mycobacterial properties due to bacilli entrapment in the extracellular milieu [10]. Given the ability of GO to reach lungs when injected intravenously in mice, we see GO as a potential candidate to treat a pulmonary disease as TB [12].

In this study, we have evaluated GO co-administration with INH, one of the most efficient drugs against susceptible TB, and two second-line drugs as AMK and LZD [15,16], in relevant models of in vitro *Mtb* infections.

LZD is a member of the oxazolidinone antibiotic class and inhibits protein synthesis by binding the 23 S subunit of the bacterial ribosome [28]. In axenic culture LZD showed a bacteriostatic effect against *Mtb* and despite the modest activity in murine models [29,30,31,32], a clinical trial showed that LZD was effective in achieving culture conversion in treatment-refractory XDR-TB patients [17]. For these reasons, LZD is widely used in the treatment of MDR-TB patients [15,16,17].

We have shown that not all drugs tested maintain their anti-mycobacterial activity when co-administrated with GO. Combined treatment of *Mtb* with GO and LZD results in enhanced inhibition of mycobacteria growth in axenic culture compared to LZD alone. Conversely, this synergistic antibacterial effect was not measured when *Mtb* was exposed to GO and AMK and GO and INH. Interestingly, interaction of these drugs with the GO surface plays a key role in this process. In fact, the strong interaction between INH and AMK with the GO surface, likely through the -COOH and -NH2 groups available on GO and drugs, respectively, may destabilize the nanomaterial. Conversely, LZD is not adsorbed on the GO surface, probably due to the lack of aminic groups. Indeed, the administration of GO with LZD enhanced LZD activity, up to half the MIC point. We hypothesize that GO trapping may favor the interaction between LZD and mycobacterial cell wall providing a higher drug internalization. This addictive effect was also observed in *Mtb* infected macrophages and targeted mainly extracellular bacilli.

GO-mediated ROS stimulation may enhance the bactericidal activity of these molecular species, that are known to have a key role during *Mtb* infection [33] and maximize the LZD antimycobacterial activity. Indeed, ROS molecules, H_2_O_2_ in particular, exert an important signaling function that influence the response of nearby cells [34]. Interestingly, bacterial membranes exhibit a limited permeability to H_2_O_2_ [35] and GO could modify ROS internalization through the mycomembrane, destabilizing the mycobacterial homeostasis and consequently facilitating the activity of the LZD. On the other hand, GO could perturbate eukaryotic cell membranes and enable passive uptake of this drug, estimated to be less than 10% [36], in the infected cells. Further studies will be needed to shed light on this mechanism.

Among the most important pathogenic properties of *Mtb* is its ability to entry, resist and replicate intracellularly in macrophages [37]. Hence, the trapping properties of GO, associated or not with drug as LZD, may be seen as poorly relevant in vivo. However, we know that *Mtb* replicates reside extracellularly in different pathogenetic steps of TB. For instance, *Mtb* can replicate extracellularly, eventually forming clumps, in tissue and even in solid granulomas [38]. Most importantly, in caseous and necrotic granulomas, large loads of extracellular *Mtb* are found in the caseous milieu where these bacilli further trigger inflammation to promote tissue damage [39,40]. A combination of GO with LZD can reduce the extracellular bacteria loads that may rapidly curb, thanks also to the entrapment properties, inflammation and therefore tissue damage.

This GO-LZD combination may be useful, especially for MDR-TB, where the property of the GO are added to LZD activity. LZD is indeed preferred as a treatment for these cases together with bedaquilne and a fluoroquinolone [15,16,17].

In conclusion, GO nanomaterials represent a strategy useful to change pathogens–host cells interactions. GO-LZD combination could be a potential formulation to enhance the mycobacterial extracellular and intracellular milieu, even using LZD concentrations below the MIC point thanks to a synergic effect between GO trapping, GO-mediated ROS generation and LZD antimicrobial effect. Future studies will deepen GO potentiality as a delivery platform for anti-TB drugs. On one side, it will be important to characterize the biological and molecular mechanism responsible for the enhanced antimicrobial activity of the GO-LZD formulation over LZD alone. Moreover, the properties of GO prepared by different methods can vary and the content and type of oxygen functionalities vary accordingly [41,42]. Therefore, it will be important to perform in vivo models of infection and prior clinical translation, such that a standardized large-scale production of the nanomaterial is obtained. Different functionalized nanomaterials and diverse formulation of nanomaterial and existing drugs should be assayed to evaluate cytotoxicity.

In parallel, experimental evidence in relevant preclinical animal models will be useful to assess the clinical potential of this formulation. Evaluation in the mouse model of TB of regimens based on LZD failed to show relevant anti-TB activity that was instead later demonstrated in human clinical trial [17]. Evaluation in larger rodents, such as rabbits, that develop caseous necrosis similar to that observed in humans, may represent the most appropriate model to assess the activity of GO-LZD combination.
